# Dyslipidaemia as a risk factor in the occurrence of stroke in Nigeria: prevalence and patterns

**DOI:** 10.11604/pamj.2016.25.72.6496

**Published:** 2016-10-04

**Authors:** Michael Adeyemi Olamoyegun, Akinyele Taofiq Akinlade, Michael Bimbola Fawale, Anthonia Okeoghene Ogbera

**Affiliations:** 1 Department of Internal Medicine, Endocrinology, Diabetes & Metabolism Unit, LAUTECH Teaching Hospital, and College of Health Sciences, Ladoke Akintola University of Technology, Ogbomoso, Oyo State, Nigeria; 2 Department of Medicine, General Hospital, Odan, Lagos, Lagos State, Nigeria; 3 Department of Medicine, Obafemi Awolowo University Teaching Hospital Complex, and Obafemi Awolowo University, Ile-Ife, Osun State, Nigeria; 4 Department of Medicine, Lagos State University Teaching Hospital, Ikeja, Lagos state

**Keywords:** Stroke, dyslipidaemia, risk factors, prevalence, patterns

## Abstract

**Introduction:**

Stroke is a major public health problem worldwide. Hypertension, diabetes mellitus, dyslipidaemia and smoking are some of the common modifiable risk factors in the occurrence of stroke. Therefore, this study was designed to assess the prevalence and patterns of dyslipidaemia among individuals with acute stroke.

**Methods:**

This is a retrospective descriptive cross-sectional study, carried out in the Departments of Medicine at the LAUTECH Teaching hospital, Ogbomoso and General Hospital, Orile-Agege, Lagos, South-West, Nigeria, over a 18-month period between September 2012 and February 2014. One hundred and six (106) patients with acute stroke confirmed with computed tomography (CT) brain scan were recruited. Clinical features, risk factors, lipid profiles and stroke patterns were identified.

**Results:**

Mean age was significantly higher in ischaemic stroke compared to haemorrhagic (64.08±10.87 Vs, 56.21±12.38years, p=0.001). There was slight male preponderance in both stroke types (1.3:1). Out of 106 patients, 65 (61.3%) had ischaemic stroke, 38 (35.8%) intracerebral haemorrhage and 3 (2.9%) with subarachnoid haemorrhage. Dyslipidaemia is the most frequent risk factor (85.9%), followed by hypertension (66.0%) and diabetes mellitus (15.1%). Dyslipidaemia was significantly higher in the ischaemic stroke compared to haemorrhagic. Reduced HDL-cholesterol is the most prevalent fraction of lipid abnormalities (74.5%).

**Conclusion:**

Dyslipidaemia, particularly low HDL-C, was the most frequent risk factor in our patients with stroke. Hence, prevention of dyslipidaemia as well as other risk factors is key to reducing the burden of stroke in our country.

## Introduction

Stroke is defined as a rapidly developed global or focal neurological deficit lasting more than 24 hours or leading to death with no apparent cause other than vascular origin [1]. It is the leading cause of mortality and long-term disability worldwide with up to one of every six survivors remaining permanently disabled [2]. In industrialised countries, stroke accounts for about 10% of all deaths [3]. In Nigeria, the frequency of stroke hospitalization ranges from 0.9 to 4.0%, 0.5 to 45% of neurological admissions, 3.7% of emergency medical admission, and found to be eighth leading cause of death [4, 5]. Of all strokes, 88% are classified as ischaemic, and remaining 12% comprise of haemorrhagic, either subarachnoid (9%) or intracerebral (3%) [6]. Hypertension (HTN), atrial fibrillation, other cardiac diseases, dyslipidaemia, diabetes mellitus (DM), cigarette smoking, physical inactivity, carotid stenosis, and transient ischemic attack (TIA) are all modifiable risk factors that predispose to stroke [7]. Dyslipidaemia is the presence of abnormal levels of lipids or lipoproteins in the blood, which includes elevated total cholesterol (TC), elevated low density lipoprotein cholesterol (LDL-C), elevated triglycerides (TG) and reduced high density lipoprotein cholesterol (HDL-C) [8]. Dyslipidaemia, especially elevated levels of LDL-C is a major risk factor for cerebral infarction by promoting formation of atherosclerosis [9, 10]. Atherosclerosis is the most important pathologic event among cardiovascular (CV) diseases and dyslipidaemia is one of the most important risk factors for developing atherosclerosis. Stroke has been shown to be related to blood lipid levels [11, 12]. Hence reducing LDL level to target can significantly reduce the risk of cerebral infarction and stroke [13]. However, the role of dyslipidaemia in the pathogenesis of haemorrhagic stroke is not clear, it has been suggested that elevated level of LDL might increase the risk of haemorrhagic stroke [14]. In Nigeria and, indeed, sub-Saharan Africa, there is inadequate data on the prevalence and patterns of lipids in relation to stroke phenotype, hence this study was aimed to ascertain the association between dyslipidaemia and stroke subtypes among patients who presented in our centres.

## Methods

This was a retrospective descriptive study carried out on patients with history and examination in keeping with acute stroke during an 18-month period between September, 2012 and February, 2014. Patients were recruited from the departments of Medicine of LAUTECH Teaching Hospital, Ogbomoso and General Hospital, Orile-Agege, Lagos. Both hospitals are located within South-West, Nigeria. The study protocol was approved by the Ethics and Research Committee of the LAUTECH Teaching Hospital. Patients under drugs that treat dyslipidaemia were excluded. Stroke was defined and categorized into hemorrhagic and ischemic stroke based on brain computerized tomography (CT) scan findings. Fasting lipid profile including total cholesterol (TC), low density lipoprotein cholesterol (LDL-C), high density lipoprotein cholesterol (HDL-C), and triglycerides (TG) levels expressed in mg/dL were also obtained from the records. Only patients whose blood samples for lipid profile were collected within 72 hours of presenting in the hospital were recruited into the study. Lipid abnormality was defined as raised TG level ≥ 150mg/dL, reduced HDL-Cholesterol - <40mg/dL in males and < 50mg/dL in females and TC level ≥ 200mg/dL [15]. The atherogenic index and lipid ratios were calculated using the following established formulas [16, 17].

Atherogenic index of plasma (AIP) = Log [TG/ HDL_c_

Castelli Risk Index − I (CRI'>TG/ HDL_c_

Castelli Risk Index − I (CRI- I) = TC/ HDL_c_

Castelli Risk Index − II (CRI- II) = LDL_c_/ HDL_c_

Atherogenic co − efficient (AC) = TG - HDL_c_ / HDL_c_

CHOLIndex = LDLc − HDLc [TG < 400] = LDLc − HDLc + 1/5TG [TG > 400 >TG > 400]

The following are the abnormal values of atherogenic index of plasma, lipid ratios, and CHOLIndex for cardiovascular risk: AIP > 0.1, CRI−I > 3.5 in males and >3.0 in females, CRI−II > 3.3, AC > 3.0 and CHOLIndex > 2.07 [16, 18–20].

### Data analysis

The data obtained from the study was analyzed using Statistical Package for Social Sciences version 16 (SPSS vs. 16) for Windows. Descriptive statistics were presented as mean ± standard deviation (SD), range and frequency (% values). For comparisons between two groups, independent sample t test was used for normally distributed numerical variables. After performing homogeneity of means test, Oneway Anova was used for comparisons between multiple independent groups. Chi-square test was used for categorical variables. Statistical significance was set at P <0.05. Multivariate logistic regression analysis was done to determine the factors that predict or differentiate subtypes of stroke.

## Results

**Figure 1:**
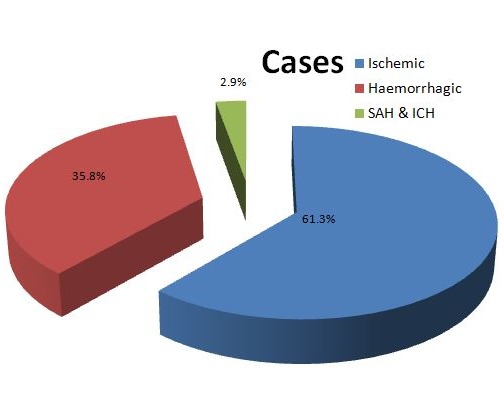
Summary of the number of stroke cases

**Figure 2:**
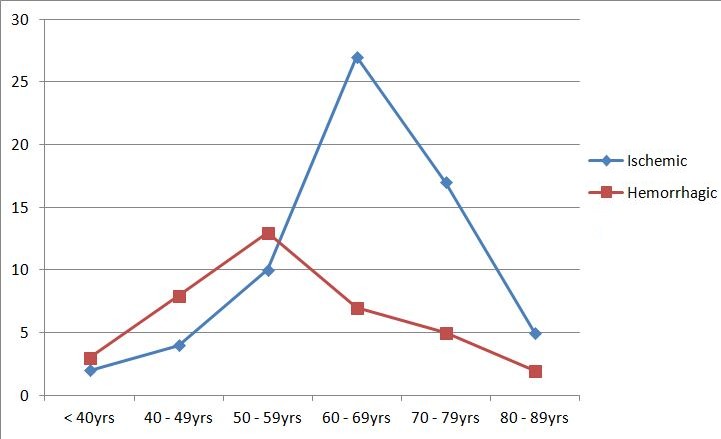
Age distribution of the stroke subtypes

**Figure 3:**
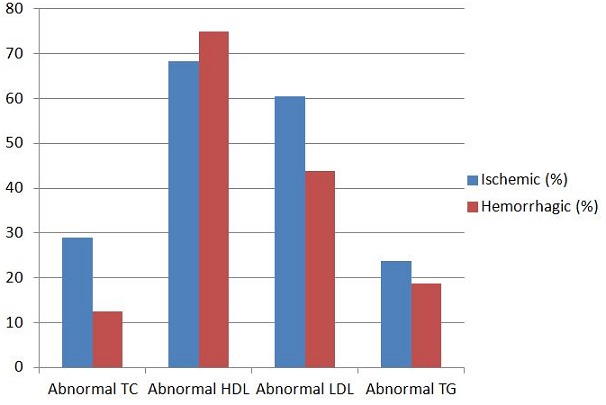
Abnormal Lipid profile among the two major subcategory of stroke patients

A total of 106 patients participated in our study comprising of 61 (57.5%) males. The mean age was 56.21 ± 12.38 and 64.08 ± 10.87 years for the hemorrhagic and ischemic stroke patients respectively. Sixty-five (61.3%) of the patients had Ischemic stroke, 38 (35.8%) hemorrhagic stroke, while 3 (2.9%) patients had subarachnoid haemorrhage (Figure 1). The history of diabetes, hypertension, angina pectoris and intermittent claudication were commoner in patients with ischemic stroke compared with hemorrhagic stroke as shown in Table 1. Of all the admission signs and symptoms, headache, vomiting and loss of consciousness were significantly higher among the hemorrhagic stroke patients compared to those with ischemic stroke (23 vs. 12; p=<0.001, 18 vs. 9; p=<0.001, 21 vs. 20; p=0.013) respectively. Incidence of stroke increases with age (Figure 2). The incidence of ischemic stroke peaks in the 60 - 69 years age group while it peaks in the 50 - 59 years age group for the hemorrhagic stroke. In both groups, about 60.4% of the patients were more than 60 years old. Figure 3 shows the abnormal lipid profiles in the two major subcategories. Table 2 shows the clinical and biochemical characteristics of the patients. The admission systolic blood pressure (SBP) was significantly higher in the hemorrhagic stroke patients (183.03±33.99 vs. 159.46±31.87; p=0.001). All the other parameters were higher in the ischemic stroke patients than in the hemorrhagic stroke patients apart from the admission diastolic blood pressure (DBP), although this difference was not statistically significant. On evaluation of the atherogenic index of plasma and the lipid ratios, they were higher in the ischemic stroke patients, although this difference was not statistically significant (Table 2). On logistic regression, only Siriraj score independently differentiated ischaemic stroke from haemorrhagic stroke (OR=6) (Table 3).

**Table 1:** The admission presentation of the stroke patients

Variable^+^	Hemorrhagic (n=38)	Ischemic (n=65)	*p* value
**Age (years)**	**56.21±12.38**	**64.08±10.87**	**0.001**
**Sex**			0.287
Male	24	36	
Female	14	29	
History of HTN	25	45	0.441
History of diabetes	5	11	0.417
History of IC	0	2	0.396
Headache	23	12	<0.001
Vomiting	18	9	<0.001
Loss of consciousness	21	20	0.013
Seizure	2	11	0.075
Drowsy	8	9	0.247

+ 3 patients had sub–arachnoid hemorrhage (SAH)

**Table 2:** A comparison of clinical and biochemical parameters of the patients with Hemorrhagic and Ischemic stroke

Variable	Hemorrhagic	Ischemic	*p* value
Admission SBP (mmHg)	183.03±33.99	159.46±31.87	0.001
Admission DBP (mmHg)	106.18±20.51	96.66±27.30	0.065
HTN	37	54	0.054
Admission pulse pressure (PP)	76.84±30.02	62.80±24.82	0.019
Admission mean arterial pressure (MAP)	131.80±21.57	117.59±26.43	0.008
Admission blood glucose (mg/dL)	138.11±53.01	149.82±95.10	0.495
TC (mg/dL)	165.06±36.66	193.55±71.91	0.140
LDL-chol (mg/dL)	104.18±35.78	129.74±71.13	0.179
HDL-chol (mg/dL)	40.69±13.76	41.87±15.33	0.791
TG (mg/dL)	106.56±44.76	126.87±73.04	0.308
Dyslipidaemia	31	60	0.475
CRI-I	4.26±0.85	5.37±2.91	0.143
CRI-II	2.66±0.78	3.71±2.42	0.098
AC	3.26±0.85	4.37±2.92	0.143
CHOL-Index	1.64±0.85	2.30±1.92	0.192
AIP	0.62±0.10	0.68±0.20	0.253

**Table 3:** Logistic regression of the predictors of stroke subtype

Variable	OR	95% CI	*p*–value
Age	1.055	985 - 1.129	0.125
Admission SBP	1.018	983 - 1.053	0.323
CRI_I	715	146 - 3.514	0.680
CRI_II	1.611	271 - 9.567	0.600
PP	958	911 - 1.007	0.093
Siriraj score	6.024	1.383 - 26.244	0.017

## Discussion

The prevalence and pattern of dyslipidaemia among stroke patients in Nigeria has not been sufficiently studied, justifying this study. This retrospective study showed that the incidence of stroke increased with age and the peak incidence for ischaemic and haemorrhagic strokes were in the 7^th^ and 6^th^ decades respectively. This shows that older patients developed more ischaemic stroke compared to haemorrhagic stroke. This trend suggests that increasing age (or risk factors associated with age) increases the risk of an infarction more than it does haemorrhagic stroke. This is similar to findings in other studies in Nigeria [21, 22] and in the United State of America [23]. The male to female sex ratio of 1.3:1 is in keeping with other studies that reported male preponderance [21, 22]. The male sex has been listed as a risk factor for stroke [24]. There was a higher incidence of ischaemic stroke (61.3%) than haemorrhagic stroke (35.8%). This is in keeping with a study in South-West Nigeria which also reported higher prevalence of ischaemic stroke compared to haemorrhagic stroke [25] and 65% versus 31% reported by Eze et al [21]. Sixty-five (61.3%) had ischaemic stroke compared to 39 (35.8%) and 3 (%) with haemorrhagic stroke and SAH respectively. This is similar to Desalu et al [25] which reported 64%, 31% and 3% for ischaemic, haemorrhagic and SAH respectively.

In our study, we observed that, besides age, dyslipidaemia was the most frequent risk factor (85.9%), followed by hypertension (66.0%) and hyperglycaemia (15.1%), constituting major risk factors in our stroke patients. Khan et al [26] found hypertension (65%), dyslipidaemia (32.7%), DM (31.3%) and smoking (32%) as major risk factors for stroke. The prevalence of hypertension reported in this study is lower than 86.5% reported by Desalu et al [25] in South-West Nigeria and 76% reported by Eze et al [21] in Abakaliki, South-Eastern Nigeria. The prevalence of DM (15.1%) in this study is also lower than 23.8% reported in South-West, Nigeria by Desalu et al [25] where DM was ranked second, but higher than 8% reported in North-Eastern Nigeria by Ogun et al [27]. The presence of DM was significantly higher among those with ischaemic stroke compared with haemorrhagic stroke. DM is a major risk factor for the development of atherosclerosis, an important pathologic mechanism in the development of ischaemic stroke. Excess risk of stroke in patients with DM is about 2-4 times higher compared with individuals in a general population without diabetes [28].

We found that, overall, the prevalence of dyslipidaemia in our study sample as whole was 85.9%. The prevalence of dyslipidaemia in ischaemic stroke (92.3%) was significantly higher than in haemorrhagic (81.6%). Mirghan & Zein [29] found that the prevalence of dyslipidaemia in ischaemic stroke patients in Emarates [30] was 65% which is lower than in our finding. These differences in the prevalence of dyslipidaemia in different places may be attributed to the differences in genetic pattern, cultures, lifestyles and dietary habits. The high prevalence of dyslipidaemia in our study may also be attributed to the possibility of insulin resistance in our subjects, since most of our patients are hypertensive (66%).

As regard the patterns of dyslipidaemia in our study, low HDL-C is the most prevalent pattern in our study (74.5%), followed by elevated LDL-C (7.5%), high TC (5.7%) and elevated TG (4.7%). There was no significant difference in the pattern of lipids among ischaemic stroke compared to haemorrhagic stroke, although all lipid parameters were higher in ischaemic stroke compared to haemorrhagic stroke. This is in conformity with other Nigerian studies [31–34], that also found a very high prevalence of low LDL-C in their participants, as the most prevalent lipid abnormality. Our findings, however, differ from those in Caucasian populations, where reduced HDL-C was said to be uncommon in ATP III [15]. It is also different from a previous study by Zhang et al [35] that found high LDL-C as the most prevalent dyslipidaemia among ischaemic stroke. Low levels of HDL-C has been shown to be associated with increased risk of stroke especially ischaemic stroke as reported by several studies [36, 37] that found an inverse association between HDL-C and ischaemic stroke risk in both gender.

Regarding lipid ratios, both CRI-I (TC/HDL-C) and CRI-II (log [TC/LDL-C]), were significantly higher among patients with ischaemic stroke compared to those with haemorrhagic. Zhang et al [37] found a positive association between elevated CRI-I and ischaemic stroke.

## Conclusion

Evidence from this study suggests that dyslipidaemia, followed by hypertension and diabetes, is the most frequent stroke risk factor in our sample. Although elevated LDL-C is usually the lipid fraction implicated in the pathologic mechanism of stroke, our study showed a significant proportion of our study subjects had reduced HDL-C. Hence, control of dyslipidaemia should be targeted in the primary prevention of stroke in order to reduce the burden of stroke in our country.

### What is known about this topic

- Dyslipidaemia is one of the modifiable risk factors for the development of stroke;
- Elevated low density lipoprotein cholesterol (LDL-C) is the lipid subtype most implicated in the pathologic mechanism of stroke.

### What this study adds

- Dyslipidaemia is the most frequent risk factor (85.9%) found in those with stroke, followed by hypertension (66.0%);
- Reduced high density lipoprotein cholesterol (HDL-C) is the most prevalent fraction of lipid abnormalities (74.5%), followed by elevated LDL (7.5%), elevated total cholesterol (5.7%) & high triglycerides (4.7%);
- Castelli risk index (CRI-I)-TC/HDL and Castelli risk index-II (Log [TC/LDL-C]) significantly higher among patients with ischaemic stroke compared with those with haemorrhagic subtype.
